# Influence of upscaling on identification of reservoir fluid properties using seismic-scale elastic constants

**DOI:** 10.1038/s41598-019-49559-2

**Published:** 2019-09-10

**Authors:** Shengjie Li, Daxing Wang, Mengbo Zhang

**Affiliations:** 1 State Key Laboratory of Petroleum Resources and Prospecting, China University of Petroleum (Beijing), CNPC Key Laboratory of Geophysical Exploration, Beijing, 102249 China; 2 Exploration and Development Research Institute of PetroChina Changqing Oil Field Company, Xi′an, 710018 China; 3 National Engineering Laboratory for Exploration and Development of Low-Permeability Oil & Gas Fields, Xi′an, 710018 China

**Keywords:** Geophysics, Seismology

## Abstract

Elastic constants derived from seismic-scale measurements are often used to infer subsurface petrophysical properties based on rock-physics relationships established from either theoretic model or core-scale measurements. However, the spatial heterogeneity of rock physical properties at the local scale has a significant impact on this relation. To understand this problem, we built a scaled physical model comprised of artificial porous layers with different pore fluids. After conducting a two-dimensional marine seismic survey over the physical model, the physical modeling data ware then used to retrieve the elastic constants of the layered package. The seismic-scale results reveal that the identification of reservoir fluid properties is improved using elastic constants that is more sensitive to pore fluid properties. The results of numerical simulations show that Lamé moduli provide more insight into rock properties and pore-fluid contents than P-wave impedances, and that the relationship between the upscaled elastic constants and the effective fluid bulk moduli at the seismic scale is usually not perfectly preserved at the reservoir scale. To interpret seismic-scale elastic constants for petrophysical properties, the rock physics relationship need to be carefully calibrated. The findings will help us understand the upscaling of rock-physics transform, which will improve the accuracy of geological property predictions from seismic-scale elastic constants.

## Introduction

Seismic methods are among the principle tools used in oil and gas exploration, CO_2_ sequestration monitoring, mineral exploration and other areas. Seismic signatures are important sources of information on the physical properties of subsurface and commonly used to infer reservoir fluid properties, based on the relationship between undrained elastic constants and pore-fluid bulk modulus established at local scale. However, one issue with this is that the relationship between undrained elastic constants and pore-fluid bulk modulus established in the laboratory or well can be different and may not be directly used in interpreting seismic data whose spatial scale is much coarser. The commonly used theory describing wave propagation in fluid-saturated media is Biot’s equation of poroelasticity^1^, which forms the basis for relating poroelastic constants with the fluid bulk modulus for homogeneous media. The prediction of Biot and Gassmann’s equations^2^ have been confirmed by both laboratory and field experiments^3–5^; however, this theory has been limited by the explicit assumptions of homogeneous fluids and solid phases, and it is often used to establish the relationship between undrained poroelastic constants and the pore-fluid bulk modulus for centimeter-scale rocks or porous layers (such as reservoirs). Laboratory measurements have shown that the Biot-Gassmann equation may have different behavior for heterogeneous systems^4,6^. Determining the valid relationship between poroelastic constants and the effective fluid bulk modulus of heterogeneous systems at seismic scale remains challenging.

Stratified poroelastic materials are typical heterogeneous composites. Much effect has been devoted to estimating the effective properties of heterogeneous composites in the geophysical literatures^7–14^. Multiple methods exist for the upscaling of elastic properties from finer-scale measurements. Gelinsky & Shapiro^9^ derived the analytical expressions for effective stiffness tensors of a layered porous package at no-flow and quasi-static conditions. Berryman^12^ deduced the compliance form expression for stratified porous media under the same assumptions used in Gassmann’s equations. Wollner & Dvorkin^15^ discussed a method for estimating the effective solid bulk modulus and fluid bulk modulus of a layered poroelastic package from fine-scale measurements. Recently, authors have been investigating the rock physics transform at different scales^15,16^. For example, Moysey & Knight^17^ show that the field-scale rock physics relationships may not be consistent with those established on core-scale measurements. Berryman^12^ shows that Biot’s equation is not the correct equation at the macro-scale, when there is significant heterogeneity in the medium’s fluid permeability. Dvorkin & Wollner^18^ show how to deal with the issue of upscaling rock-physics laws by using synthetic examples and conclude that the rock-physics transform between the elastic constants and the petrophysical properties established on the borehole-scale data approximately holds at the seismic scale under certain conditions. Wollner & Dvorkin^19^ explore the seismic-scale dependence of the effective fluid bulk modulus upon water saturation and find that this relation trends toward an arithmetic average of the individual bulk moduli of the pore-fluid phases. However, the fluid-dependence of poroelastic constants at the seismic scale is poorly understood. This is due to the fact that the direct measurement of seismic-scale relationship between undrained elastic constants and effective fluid bulk moduli is a hard task if possible, and it is very difficult to verify this relationship at the seismic scale with elastic constants deduced from field-scale data. In addition, there are few literatures about laboratory experiments to study seismic-scale relationship between undrained elastic constants and effective fluid bulk moduli for heterogeneous media.

Physical modeling has been a useful tool to better understand the relationship between seismic responses and scaled porous layers in laboratory analogs of field reservoirs. The reflection amplitude variation with increasing offset acquired by physical modeling experiments can be used to retrieve elastic constants, which useful for discriminating lithology types and identifying pore fluids^20,21^. In this study, physical modeling and numerical simulations are used to explore the effect of upscaling on the fluid dependence of undrained elastic constants at the seismic scale. The present work is restricted to heterogeneous layered systems arising from the layering of homogeneous porous materials. We define the seismic scale (>tens of meters,) and the reservoir scale (several centimeters to tens of meters) as two distinct scales of interest. It is well known that mesoscopic heterogeneities can cause dispersion and attenuation of seismic waves due to sub-wavelength scale wave-induced fluid flow^22–26^. To emphasize the poroelastic behavior of layered porous media, our focus here is on the seismic-scale dependence of poroelastic constants upon the effective fluid bulk moduli of heterogeneous systems at no-flow limits. Our approach to the derivation of rock-physic relationships at the seismic scale involves two-dimensional physical modeling and numerically forward simulation using one-dimensional earth models. First, physical modeling, which is believed the best way to mimic real wave propagation in fluid-saturated porous media, is used to produce real reflection data, which are then inverted to the seismic-scale elastic constants. Next, the physical properties of each layer in the physical model, such as porosity, bulk moduli of pore-fluid, drained and undrained rocks, are upscaled using different upscaling methods. Finally, the seismically inverted elastic constants are compared with those obtained from numerical simulations. The results of this study can potentially improve the quality of interpretation of seismic-scale elastic constants for rock physics properties.

## Results

### Physical model

**Figure 1:**
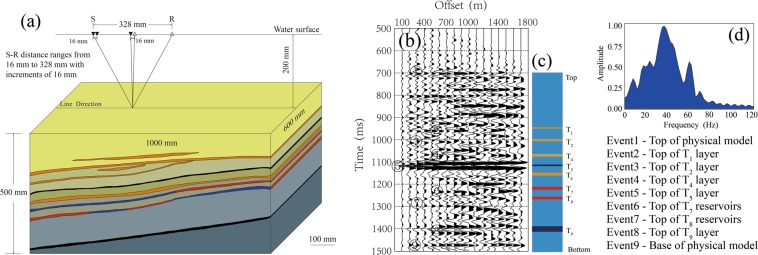
(**a**) A schematic three-dimensional model used for physical modeling. (**b**) The collected CMP gather with major reflected events calibrated by the stratigraphic section (**c**), and the amplitude spectrum of the target.

To obtain closer to field seismic signals, a scaled physical model with dimensions of 1000 × 600 × 500 mm was constructed. Figure 1(a) shows a schematic diagram of our physical model, which simulates a real-world onshore lithostratigraphic hydrocarbon reservoir. Such a setup may be relevant to depositional settings such as fan delta front regions, where high quality hydrocarbon reservoirs are surrounded by tight rocks. The physical model consists of 19 layers; there are 10 tight layers simulating mudstone formations and 9 simulated sand layers sandwiched between the mudstone layers. These layers are composed of the mixtures of epoxy resin and silicone rubbers (except for the seventh and eighth sand layers, hereafter refer to as T_7_ and T_8_), as is typical for such physical models. Hence, these layers can be treated individually as an isotropic homogeneous medium. The T_7_ and T_8_ layers are made up of synthetic, consolidated porous materials composed of the mixtures of quartz grains and resin (Supplementary Fig S3). The physical properties of all layers were precisely measured using ultrasonic transmitting techniques (Supplementary Information). The measured properties for all solid layers (Supplementary Table S2) are used as inputs for the numerical experiments. T_7_ layer was made up of two porous sandstone bodies; one filled with air, and one filled with water. The T_8_ layer was made up of four sandstone bodies: two air-filled sandstones and two water-filled sandstones were arranged alternately along the layer. An impermeable thin-resin layer between the air-filled and water-filled bodies is used for separating the fluid-saturated bodies. These fluid-saturated layers are the targets of current study.

### Common midpoint (CMP) gathers

Physical modeling is an effective technique for simulating a real seismic survey in the laboratory and to obtain seismic-scale elastic parameters. The reflection experiment was performed over the physical model. In the present study, the scaling factor for our modeling is 5,000; as a result, experimental units of 1 mm and 1 s represent field units of 5 m and 5 ms, respectively. During seismic data acquisition, the scaled physical model was submerged in the water tank, far from the walls of the tank, so as to avoid interference from the reflected waves related side wall to the primary seismic signal. The source and receiver transducers were immersed in water and positioned slightly 2.0 mm beneath the water surface to mitigate the influence of ghost on primary reflections, the interference caused by ghost waves affects the amplitude information required for seismic inversion and therefore should be avoided. The source transducer produces a wavelet with a center frequency of approximately 260 kHz, and the receiver transducers collect the reflected seismic signals. A common midpoint (CMP) shooting arrangement was employed for data acquisition. The minimum offset (defined as the distance between a source and receiver) was 16 mm, the maximum offset was 328 mm. Source and receivers were moved apart at increments of 16 mm in opposite directions. A total of 241 CMP gathers were recorded along the seismic line located in the middle of the physical model (Fig. 1(a)).

A sample CMP gather from the physical model is shown in Fig. 1(b) (all units are displayed at field scale). The frequency band of acquired data ranges from 2 to 70 Hz (Fig. 1(d)). Events corresponding to the reflection from each main reflector are visible (Fig. 1(b)). Seismic reflections are calibrated by the physical model (Fig. 1(c)). Event 1 is the *P-*wave reflection from the top of the physical model. Event 9 is the reflection from the base of the physical model. The most notable event is event 4, which resulted from the larger impedance contrast between the low-velocity layer T_5_ and its surrounding mudstone layers. These strong reflections prevent the transmission of the propagating wave energy, resulting in reduced reflection amplitudes from the lower formation. Event 8 is a *P-*wave reflection from the top of the low-velocity layer T_9_. Events 5 and 9 can be considered as markers used to calibrate the relationship between other events and the corresponding formations. The events corresponding to the reflection from the top of the T_7_ and T_8_ porous layers occur at approximately 1100 ms and 1145 ms, respectively. The reflections from the bases of these layers are not visible because the layer thickness is much smaller than the scaled seismic wavelength. The *P-*wave multiples and converted wave events are visible on the section, especially on the part above event 4. The data also contain other coherent events near the T_7_ and T_8_ layers. While the primary reflections from the target are consistent among the different CMP gathers, the coherent noises signals are not. These multiple coverage data can be used to reduce the influence of non-primary reflections on the seismic amplitudes.

### Reflection amplitude compensations

**Figure 2:**
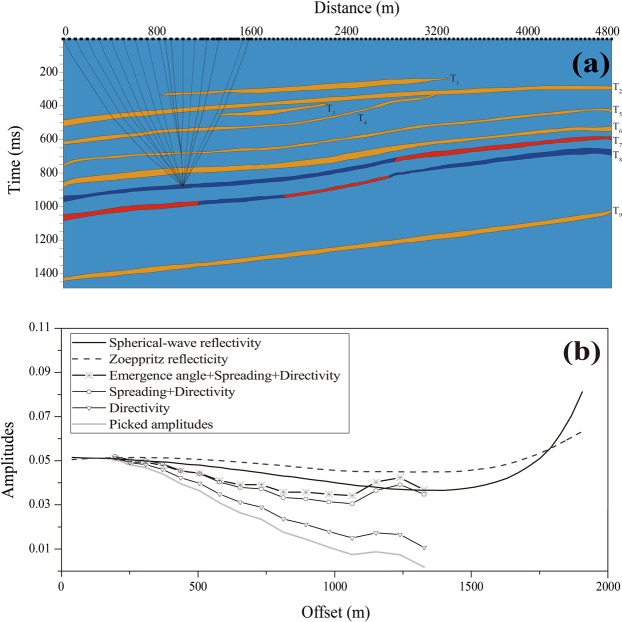
(**a**) The results of the ray-tracing calculation for determining the correction parameters. (**b**) Reflection amplitudes from the top of synthetic T_7_ layer, the raw amplitudes (grey line) were corrected by directivity of transducers, spreading, emergence angle, and compared with the plane-wave Zoeppritz solution (dashed line) and the spherical-wave solution (Black line).

To obtain seismic-scale poroelastic constants from the physical modeling data, the reflection amplitudes of the CMP gathers should first be corrected to compensate for various effects that can distort the amplitude versus offset correlations. For the physical modeling data, we corrected the amplitude of the CMP gathers for geometric spreading, emergence angles, and source-receiver directivity. In physical modeling, transducers produce a wavefield in which amplitudes are directionally biased because they have a certain diameter that is comparable with the dominant wavelength^27^. The directional behavior of transducers makes the amplitude with different offsets undesirable variations, and should be compensated prior to any seismic amplitude analysis. Following the work of Duren^28^ and Mahmoudian *et al*.^29^, directivity can be corrected by dividing measured amplitudes by the cosine of the angle between the wave propagation direction and the vertical direction. The propagation direction at the location of a transducer is called the emergence angle^21^. In the reflection amplitude correction, a ray-tracing algorithm was used to determine the primary’s raypath (Fig. 2(a)). Accurate ray tracings were obtained with the known velocities and densities of the layers in our physical model (Supplementary Table S2). By combining these results with the reflectivity calculated using Zoeppritz’s equations^30^, the compensation operator for geometric spreading can be estimated and applied accurately for the target reflector. Emergence angles for a given offset and the target depth was calculated by ray tracing through all the layers using the final angle at a receiver; the inverse of the cosine of the emergence angle can then be applied to the reflection amplitudes.

To compensate the physically modeled data, the reflection amplitudes from the top of the synthetic porous layer T_7_ were picked from the primary event on the raw data and corrected for the effects of transducer directivity, geometric spreading, and emergence angle. Figure 2(b) shows a sample reflection amplitude correction process from a CMP gather. To validate the corrected amplitude, the plane-wave Zoeppritz’s equation^30^ and the spherical wave equation^31^ were used to computer the theoretical reflection coefficients based on the model parameters across the interface and shown in Fig. 2(b). It was clearly shown that, after each correction, an incremental improvements was observed, demonstrating the effectiveness of each correction in improving the reflection amplitude quality.

### Seismic-scale elastic constants extractions

**Figure 3:**
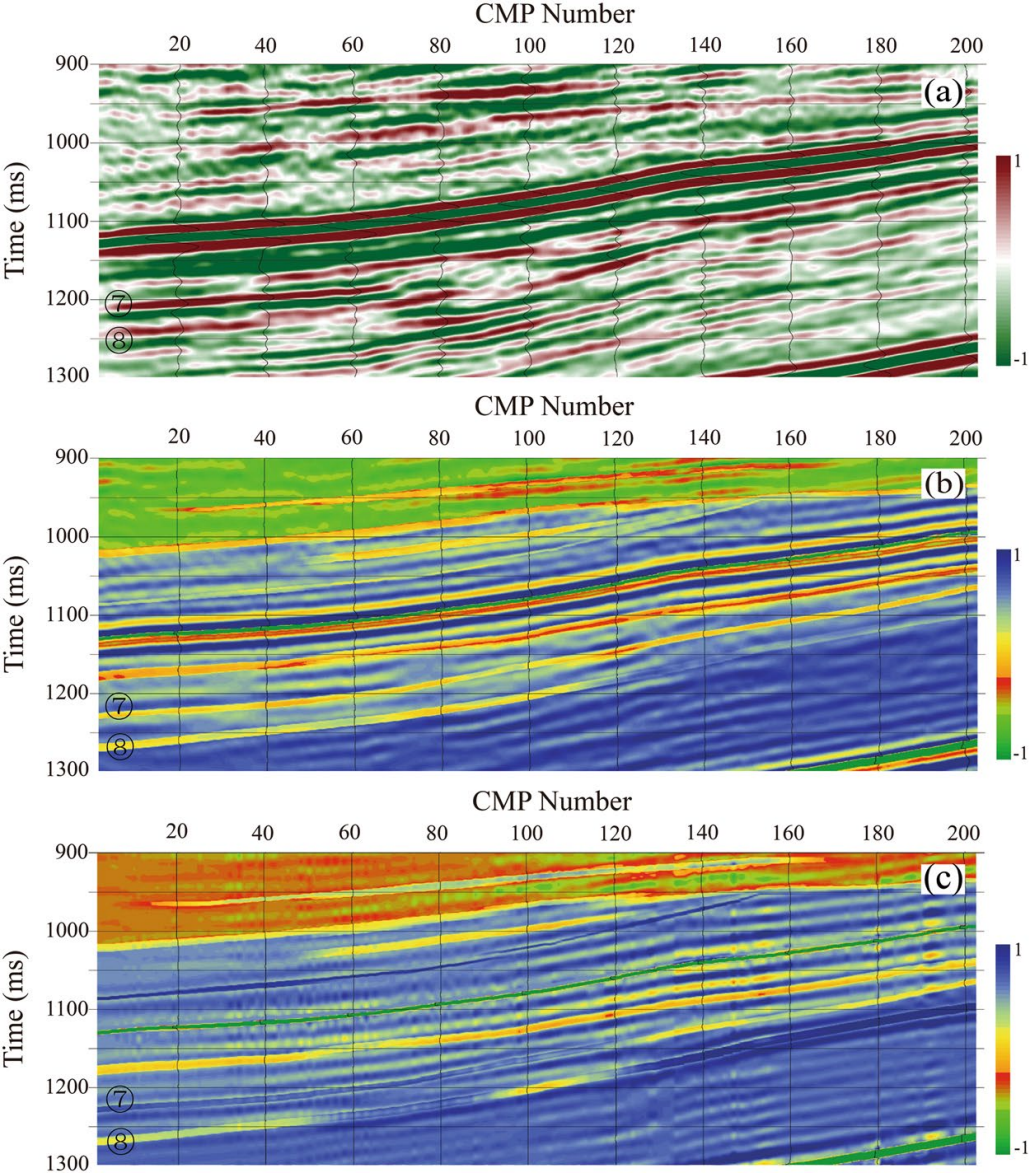
(**a**) The original stacked seismic data. (**b**) The inverted conventional P-wave impedances and (**c**) the inverted λ/μ ratio sections. The inverted results significantly improve the identification of the stratigraphy.

The offset-dependent reflection amplitude is the seismic response of elastic contrast across an interface. It provides information on the elastic constants and even pore-fluid properties of layers. Elastic constants, such as the *P-*wave impedance (I_p_), bulk modulus (K), and the first Lamé modulus (λ, or Lamé for short) of saturated rocks, are highly correlated with pore-fluid properties. For our synthetic porous layers, the air-filled layers have lower λ value than those of the water-filled layers. The second Lamé modulus (the shear modulus, *μ*) of the saturated layers is not affected by pore-fluid properties. The ratio of the Lamé modulus to the shear modulus, λ/μ, has advantages in discriminating pore-fluid properties. These elastic constants of the physical model can be retrieved using seismic inversion techniques, which are routinely used in seismic exploration. A deterministic model-based pre-stacked inversion scheme^32,33^ was used in this study because the physical modeling data are of high quality and almost free of noise. It is an ideal dataset with known stratum structures and known elastic parameters of each layer. The inversion uses a generalized linear inversion algorithm^34^ and assumes that a seismic trace is the result of the convolution of angle-dependent wavelets with reflectivity. This inversion was done iteratively by conjugate gradient methods, where the initial low-frequency models were modified until the resulting synthetic seismographs matches the seismic trace within some acceptable bounds. The result is a broadband model of λ, μ, and ρ; the resulting λ/μ is shown in Fig. 3(c). In addition, conventional seismic inversion for *P-*wave impedance was also carried out; the inversion result is shown in Fig. 3(b) for comparison. We show the seismic data in Fig. 3(a), as a reference.

From the comparison, we can see that the inverted elastic constants significantly improve the identification of the stratigraphy within the physical model. While the stacked seismic data outline the major stratigraphic boundaries along the seismic profile, it is difficult to distinguish the reflections of the air-filled layers from those of the water-filled layers in the section (marked 7 and 8 on the sections in Fig. 3). This is probably due to interference from seismic wavelets near the porous layers. The dominant frequency of the wavelet is about 45 Hz, and the average velocity of our target is about 2600 m/s. The average thickness of the synthetic porous layers is about 14.24 m, which is close to the limiting resolution, under the assumption of a one-quarter Rayleigh wavelength criterion^35^.

The inversion results indicate that the inversion removes the wavelets and efficiently improves the vertical resolution in our target zone (at times of 1060–1280 ms). However, the inverted impedance values are less useful when it comes to identify the lateral boundaries of different fluid-filled rocks in a layer. By comparing the known stratigraphy of the physical model (Fig. 3(a)), it is clear that the lateral resolving power of the inverted I_p_ (Fig. 3(b)) is less than that of the inverted λ/μ (Fig. 3(c)). In the inverted λ/μ profile, the events corresponding to the water-filled sandstone-body of the T_7_ layer are obvious, and exhibit lateral continuity at the west end of the section (at CMPs less than 125) at times of 1148–1240 ms; the event corresponding to the air-filled sandstone-body of the T_7_ layer is displayed in warmer-colors at the east end of the section (at CMPs greater than 127) at times of 1068–1140 ms. There are four fluid-filled sandstone-bodies within the T_8_ layer. The first event corresponding to air-filled body1 is sharp, and exhibits lateral continuity at the west end of the profile (at CMPs less than 46) at times of 1250–1280 ms; the second event corresponding to water-filled body1 is identifiable in the middle of the section (CMPs 47–93) at times of 1216–1248 ms. The third event corresponding to air-filled body 2 is clearly visible in the middle of the section (CMPs 95–128) at times of 1180–1210 ms; the fourth event, corresponding to water-filled body 2, is identifiable at the east end of the section (at CMPs greater than 120) at time of 1050–1178 ms. All air-filled layers are clearly visible and can be distinguished from the water-filled layers. However, these features are not as clearly resolved in the impedance results shown in Fig. 3(b); it is hard to distinguish water-filled body1 (second event) of the T_8_ layer from air-filled bodies 1 and 2 (first and third events) at the west end of the inverted I_p_ section. This may have been due to the fact that quartz is characterized by a low λ value (∼8.1 GPa) and a moderate μ value (44.4 GPa)^36^. For quartz-rich sandstones, the presence of water in the pore spaces may cause a significant increase in λ; gas-bearing rocks have lower λ. Goodway^37^ proposed that λ contains more useful information about the resistance to pressure-induced changes in volume. Hence, λ is more helpful than seismic impedance when it comes to determining pore-fluid properties. On the other hand, the relationship between the displacement and the stress of media in seismic wave propagation is determined by wave equations, which directly involve the ratio of density and modulus, not the seismic impedances. Consequently, λ and μ provide more insight into the rock properties and fluid content. The inversion results demonstrate that improved identification of reservoir boundaries is possible using elastic constant that is more sensitive to pore fluid properties. Therefore, it is important to understand the fluid dependence of elastic constants at the seismic scale.

### Effect of upscaling on undrained elastic constants

**Figure 4:**
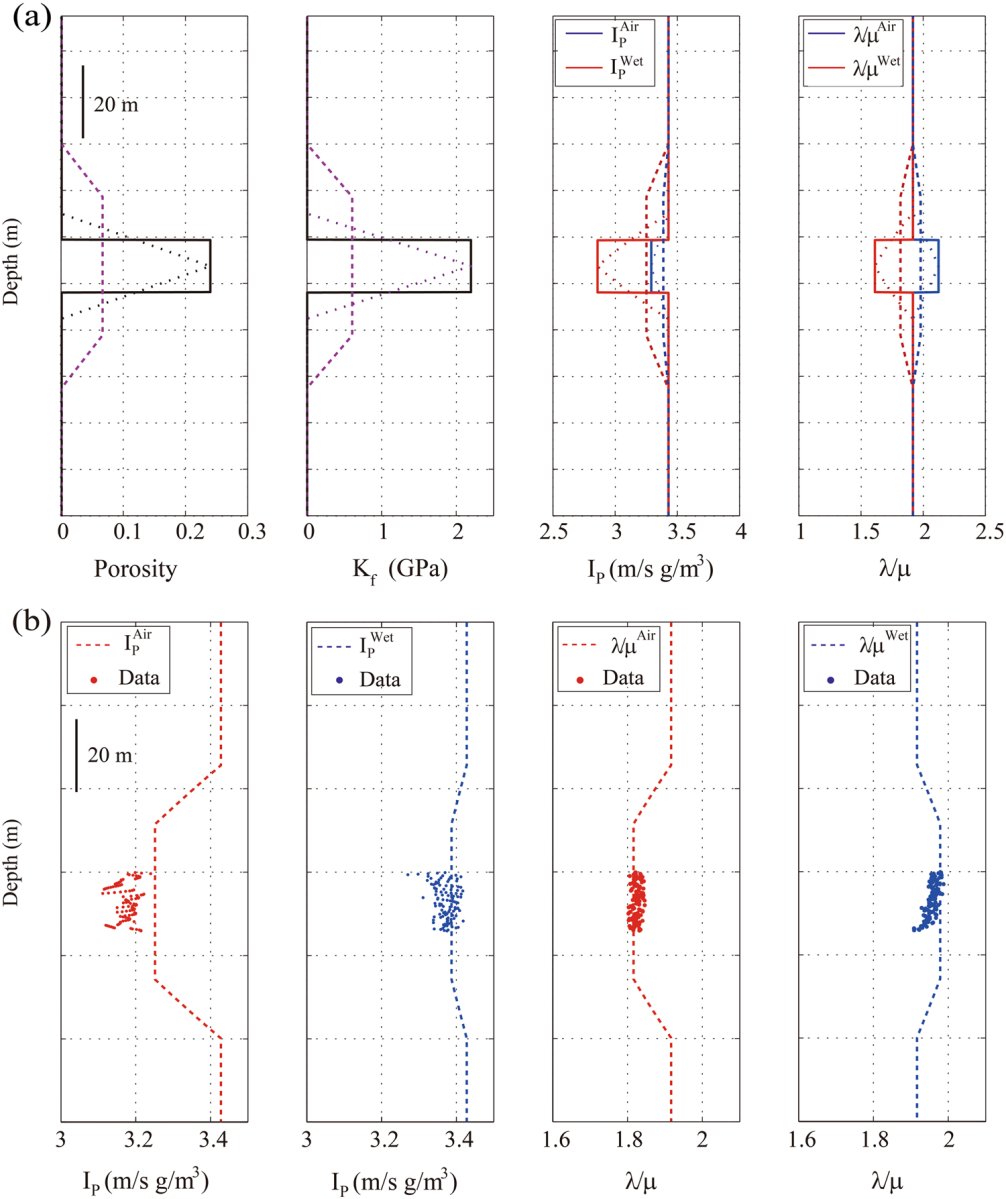
(**a**) The numerical earth model, where a 14.24 m thick porous layer located between two impermeable mudstone layers. From left to right: Porosity, fluid bulk modulus, P-wave impedance, and λ/μ ratio for air-filled and water-filled model. (**b**) The comparison of numerically upscaled P-wave impedance and λ/μ ratio (the curves) with seismically inverted ones (the scatters) for air-filled and water-filled layers.

Numerical simulations were conducted to determine the relationship between the seismic-scale elastic constants and the effective pore fluid properties so that we could quantitatively interpret the seismically inverted elastic constants. The inverted elastic moduli, such as the Lamé or the shear modulus, represent the elastic responses of the layered package at the seismic scale in the vertical direction. Hence, it is reasonable to assume that these inverted elastic moduli can also be predicted by a one-dimension upscaling method with the known elastic parameters of each layer. Because our synthetic porous layers are placed in between two impermeable resin layers, the poroelastic Backus averaging at the no-flow limit proposed by Gelinsky & Shapiro^9^ was used to estimate the seismic-scale poroelastic response of these porous layers. Poroelastic Backus averaging produces an anisotropic effective medium, where the vertical components of the upscaled poroelastic stiffnesses C_33_ and C_44_ can be converted to upscaled impedances or Lamé moduli, which can be directly compared to the seismically inverted variables. In addition, we compute the upscaled porosity and density using the volumetrically weighted average; we also compute the arithmetically volume-averaged bulk modulus of the pore fluid, which is used for reference. A representative model was designed based on the physical model (as shown in Fig. 4(a) in solid lines). The parameters used to define the earth model were listed in Table 1., and the velocity of the dry synthetic materials were measured and converted to their bulk and shear modulus of 4.6 and 2.02 GPa, respectively, and the converted undrained bulk modulus is 5.63 GPa, the average porosity and dry density are 0.24 and 1.8 g/cm^3^.

**Table 1:** Input Parameters for the Simulation in Figs 4 and 5.

	Elastic Properties of the Materials			
	Porous layer	Background	Water	Air
Bulk modulus (GPa)	7.25	6.2	2.2	0.012
Shear modulus (GPa)	7.37	2.4	0.0	0.0
Density (g cm^−3^)	1.6	1.25	1.02	0.014

For the porous layer, two cases of the earth model were run. In one run, the porous layer is fully filled with air (the red solid curve shown in third and fourth tracks); in the other, the porous layer is fully filled with water (the blue solid curve shown in the third and fourth tracks). To calculate the elastic properties of the earth models at a coarser scale, the physical properties of each layer in the earth model were upscaled using two running windows, where the first window size was equal to the length of the scaled seismic wavelength used in our physical modeling (referred to here as the seismic-scale window), and the second was equal to the thickness of the porous layer (referred to here as the reservoir-scale window), where the latter is approximately a quarter of the length of the former. The poroelastic Backus averaging results with the seismic-scale window are displayed in Fig. 4(a) as dashed lines, and those quantities upscaled using the reservoir-scale window are displayed as short dotted lines.

As expected, the results produced by different upscaling methods vary. For instance, with the seismic-scale window, the arithmetically upscaled porosity (the green dashed line on the first track in Fig. 4(a)) is reduced by 69.5% compared with the original porosity of the porous layer. The reduction of the upscaled fluid bulk modulus (the cyan dashed line on the second track in Fig. 4(a)) is almost the same as that of the upscaled porosity. However, the average reduction of the upscaled P-wave impedances or the λ/μ ratio of the air-filled porous layer is approximately 21.2% compared with their original reservoir counterparts. At the seismic scale, the relative difference between the upscaled elastic constants and the original values of the air-filled reservoirs is larger than it is for the water-filled reservoirs, with an average difference of 4%. This is likely due to the difference in the elastic contrast between the porous layer and backgrounds. The upscaled values in the center of the porous layer, determined using the reservoir-scale window, are closest to the original values for the reservoirs (Fig. 4(a)); the upscaled values are smaller than the original reservoir values when the running window is larger than the reservoir-scale window. It is interesting to note that the sensitivity of the upscaled elastic constants to pore fluid properties is different. Based on the values of the upscaled variables for air-filled reservoirs at the seismic-scale, the value of the upscaled impedance for water-filled layer increased by 3.99%, and the value of the upscaled λ/μ ratio increased by 9.58%. This means that the upscaled λ/μ ratio is more sensitive to changes in fluid properties than the upscaled impedances.

To interpret the seismically inverted data, the inverted *P-*wave impedance and the λ/μ ratio of the synthetic layers were picked and compared with the corresponding quantities of the numerically upscaled results (Fig. 4(b)). The dashed line in Fig. 4(b) represents the result of poroelastic Backus averaging using the seismic-scale window (the same as the result displayed in Fig. 4(a)), the scatters represent the seismically inverted data. It is evident that the upscaled elastic constants are comparable with the seismically inverted data; P-wave impedance errors between the upscaled parameters and the inverted data for either air-filled layers or water-filled layers is less than 3%, while the upscaled λ/μ ratios error is less than 2.6%. If we assume that the synthetic porous layers in the physical model have the same dry-frame properties, and that the elastic properties of the air-filled layer are approximately identical to those of the dry porous layer, compared to the elastic constants inverted from the air-filled layers, the inverted *P-*wave impedances increased by 6.19% for the water-filled layer and the inverted λ/μ ratio increased by approximately 7.22%.

The results of the physical modeling data and the numerical simulations indicate that poroelastic Backus averaging can be used to quantitatively analyze the effect of pore fluid properties on the upscaled *P-*wave impedances or the λ/μ ratio at the seismic-scale, and that the maximum error between the seismically inverted results and the numerically upscaled parameters is approximately 3.1%. The seismic-scale elastic constants are affected not only by the ratio of the wavelength to the thickness of a layer, but also by the elastic contrast between adjacent layers. For the poroelastic media, such as hydrocarbon-bearing reservoirs, the pore fluid properties is an important factor that affects the magnitude of the seismic-scale poroelastic constants. An important point inferred from both the laboratory and numerical simulations is that the relationship between the poroelastic constants and the pore fluid bulk modulus at the reservoir scale, as dictated by Biot-Gassmann equations, is not necessarily applicable at the seismic scale. To accurately infer the petrophysical properties of a layer from the seismic-scale effective elastic constants, the rock physics relationship at the seismic scale needs to be carefully calibrated with the aid of numerical simulations.

## Discussions

Gassmann’s equation is valid for a medium with homogeneous fluids and monomineralic solid phases. However, poroelastic Backus averaging replaces a layered medium with an effective, homogeneous anisotropic body at a coarse scale. When the Thomsen anisotropic parameter *δ* of the effective body is weak (in our case, this parameter is less than 0.02), Gassmann’s isotropic fluid substitution operation is approximately accurate^18,38^. Thus, an effective pore-fluid bulk modulus exists that will yield the undrained poroelastic response of the effective medium. To understand the seismic-scale dependence of poroelastic constants on the fluid bulk modulus, we first compared the forward-upscaled pore-fluid bulk moduli with the inverted pore-fluid modulus in an effort to analyze the magnitude of the pore-fluid bulk modulus predicted by the upscaling process. The seismically inverted elastic constants and numerical experiment results then were used to quantitatively study the fluid dependence of elastic constants at the seismic scale.

**Figure 5:**
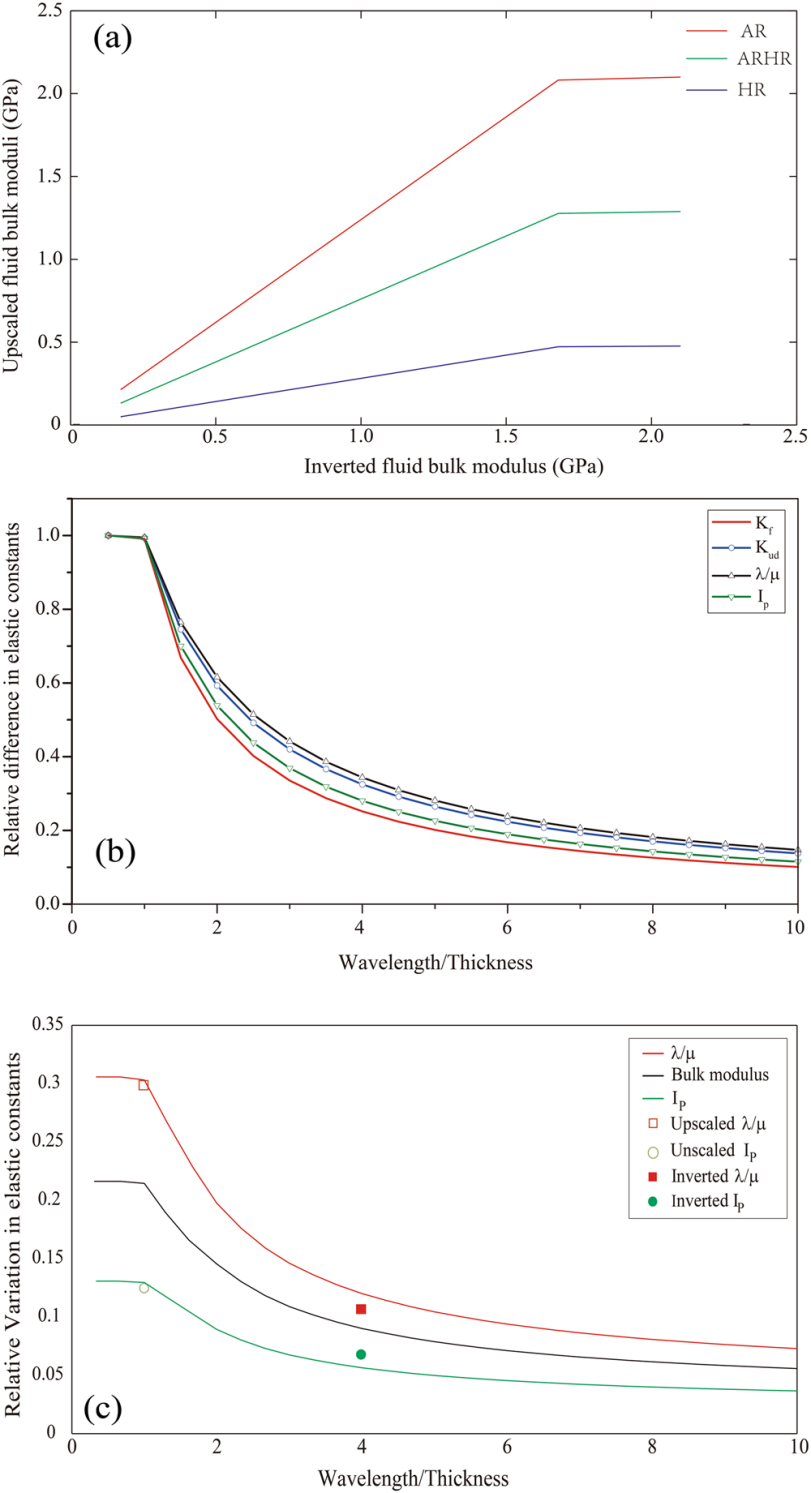
(**a**) The comparison of inverted fluid bulk modulus with the forward-upscaled fluid bulk moduli. (**b**) The relative difference in K_f_, K_ud_, λ/μ ratio, and I_p_ versus the ratio of wavelength to the thickness of the porous layer. (**c**) The comparison of the relative variation of the upscaled λ/μ ratio and I_p_ with the seismic inverted results.

To evaluate the magnitude of the upscaled fluid bulk modulus, the inverted fluid bulk modulus ${\tilde{K}}_{f}$ was obtained from the numerical simulation results by back-calculation using the inverse of Gassmann’s equation with different sizes of running windows. In addition, the effective fluid bulk moduli were upscaled from the reservoir-scale *K*_*f*_ using the arithmetic average (AR), the harmonic average (HR), and the mean (i.e. Hill’s averages) (ARHR). The inverted fluid bulk moduli from the numerical experiments were plotted against the forward-upscaled fluid bulk moduli in Fig. 5(a), where the three upscaled fluid bulk moduli are denoted as AR, HR, and ARHR. Clearly, the arithmetic average ${\tilde{K}}_{f}$ is identical to the inverted ${\tilde{K}}_{f}$ and the original value of the pore-fluid bulk modulus of the saturated layer when the running window is equal to or smaller than the thickness of the porous layer. All upscaled ${\tilde{K}}_{f}$ values will be smaller than the original value of the fluid bulk modulus as the size of the running window increases. The arithmetic average ${\tilde{K}}_{f}$ is much closer to the inverted ${\tilde{K}}_{f}$ than the other two curves. This implies that, when interpreting the seismically inverted elastic constants, we cannot arrive at the harmonic upscaled ${\tilde{K}}_{f}$, but rather at an arithmetic or Hill’s average ${\tilde{K}}_{f}$. In general, an arithmetic average ${\tilde{K}}_{f}$ is often associated with a patch saturation medium, in which patches are fully dry or fully saturated^18^. Hence, the inverted ${\tilde{K}}_{f}$ is much closer to what we would expect for a patch saturation pattern. This is consistent with the vertical arrangement of the saturated porous layers and impermeable layers in our numerical earth model.

Based on Gassmann’s equation^2^, the difference between the undrained and drained bulk moduli ${K}_{ud}={K}_{u}-{K}_{d}$, of a saturated medium is a direct measure of pore-fluid change if the only variable is related to pore-fluid properties. Accordingly, the differences between the undrained elastic constants (e.g., K_u_, I_p_, and λ/μ) and the drained elastic constants were calculated using Gassmann’s equation at the reservoir scale. Similarly, the differences between the upscaled undrained and the drained elastic constants were also estimated using poroelastic Backus average at a coarse scale. We used a relative difference, defined by the ratio of the difference between the upscaled quantities (e.g., ${K}_{ud}$) and those deduced from corresponding reservoir-scale ones, to show the behavior of upscaled elastic constants with different upscaling windows; these relative differences are plotted versus the ratio of the wavelength to the thickness of a layer (R) in Fig. 5(b).

As shown in Fig. 4(a), the physical properties of the porous layer, such as ${K}_{f}$, I_p_, and λ/μ, remain unchanged at the center of the layer when they are upscaled using a running window that is equal to or less than the thickness of the layer. These elastic constants decreased in values as the running window size increases. Hence, the reduction of the relative difference reflects the lessened sensitivity of the elastic constants to fluid properties. Also in Fig. 5b, the normalized pore-fluid bulk modulus was calculated from ${\tilde{K}}_{f}$ divided by the fluid bulk modulus of the porous layer and used for reference in our analysis. As expected, the normalized fluid bulk modulus (shown in red in Fig. 5(b)) decreases nonlinearly as the running widow size increases. At the seismic scale (R≈3.6), the normalized fluid bulk modulus decreased by 69% relative to that at the reservoir scale (R ≤ 1). The upscaled ${K}_{ud}$ obtained at reservoir scale (R ≤ 1) is equal to that of the water-filled porous layer, and 64% smaller at seismic scale than it is at the reservoir scale. The relative differences in I_p_ are smaller than those in ${K}_{ud}$, but the relative differences in λ/μ are larger than those in the ${K}_{ud}$ when the running widow is larger than the thickness of the porous layer. In our case, the relative differences of the elastic constants obey the same trend of the normalized fluid bulk modulus.

The difference between the undrained and drained elastic constants quantitatively represents the extent of variation in the elastic constants induced by pore-fluid changes. Therefore, the differences between upscaled undrained elastic constants and drained ones were calculated from our numerical experiments at a coarse scale. These differences then were divided by the corresponding drained constants (to determine the relative variation) and plotted those variations against the ratio of the wavelength to the thickness of a layer (Fig. 5(c)). Similarly, the values of the seismically inverted I_p_ and λ/μ of the air-filled and water-filled layers were picked, respectively. The mean and variance of the inverted I_p_ values are 3.17 ± 0.026 km/s g/cm^3^ for the air-filled layer and 3.34 ± 0.026 km/s g/cm^3^ for the water-filled layers. The mean and variance of the inverted λ/μ values are 1.82 ± 0.012 GPa for the air-filled layers and 1.97 ± 0.015 GPa the for water-filled layers. The average differences between the inverted I_p_ and λ/μ and the corresponding drained quantities were calculated respectively, to reduce the uncertainty caused by the lack of precise correspondence between the undrained and drained elastic constants. The relative variation of inverted I_p_ and λ/μ were plotted in Fig. 5(c) (solid symbols) for qualitatively calibrating the numerical simulation results. It is clear that the relative ${K}_{ud}$ variation decreases nonlinearly with increasing running window size, meaning that the sensitivity of the undrained bulk moduli to the pore-fluid properties is lessened. The undrained bulk modulus increased by 22.3% relative to the drained bulk modulus at the reservoir scale (R ≤ 1) due to the presence of water in the pore spaces of the porous layer. However, due to upscaling differences, the undrained bulk modulus only increased by 6.7% at the seismic scale (R≈3.6). Moreover, in our numerical examples, the sensitivity of the relative variation of λ/μ is relatively large, whereas that of I_p_ is small. For instance, the incremental increase of the undrained λ/μ values approached 9.2% relative to the drained one, but the increase in the undrained I_p_ values is approximately 4.2% at the seismic scale (R≈3.6).

This comparison shows that the poroelastic Backus upscaled curves are very close to the inverted I_p_ and λ/μ values. The relative variation of inverted I_p_ is greater than that of the upscaled I_p_, and the relative variation of inverted λ/μ is smaller than the variation of the upscaled values. These inverted quantities derived from physical modeling data are highly reliable because these relative variations or differences are relative values deduced simultaneously from the seismic dataset with the same quality. There are some discrepancies between the seismically inverted quantities and the numerically upscaled ones, suggesting that the poroelastic Backus average weakens the effect of pore-fluid on I_p_ and enhances the pore-fluid effects on λ/μ. We note that both the seismically inverted and numerically upscaled λ/μ values are more sensitive to the pore-fluid properties than I_p_ is at the seismic scale. As a result, we conclude that it is the fluid-sensitivity that enables us to distinguish the air-filled zones from the water-filled zones in the seismically inverted λ/μ profile. At the same time, the lateral boundaries of these air-filled and water-filled zones are less well resolved in the inverted I_p_ section.

## Conclusions

In this study, we collected seismic data from a scaled physical model with porous layers saturated different pore-fluids, to retrieve elastic constants variations with fluid saturated layers at the seismic scale. The results of seismically inverted elastic constants revealed that λ/μ is more sensitive to reservoir pore-fluid properties and is more helpful than the inverted I_p_ when it comes to identify fluid distribution, even for sub-resolution fluid-saturated reservoirs. This observation suggests that different elastic parameters have different sensitivities to reservoir pore fluid properties at the seismic scale, and that knowledge of the upscaling for elastic constants is essential to fully understand the fluid dependence of elastic constants at a coarse scale. Results of numerical simulation by poroelastic Backus averaging demonstrated that the fluid dependence of the elastic parameters of layers at the reservoir scale is well defined by Gassmann’s equation. However, the dependence of the undrained elastic constants, such as the bulk moduli, the Lamé moduli, and the impedances, on pore-fluid properties is different at the seismic scale, due to the difference in the methods used to calculate the effective fluid bulk modulus and the effective elastic moduli. For a saturated stratified medium, the effective fluid bulk modulus depends linearly on the local fluid properties whereas the relationship between the local and effective elastic constants is most likely nonlinear. Hence, upscaling affects the sensitivity of the elastic constants to the pore-fluid properties. λ/μ is more fluid-sensitive than I_p_ at various scales. It is this reason that the seismically inverted λ/μ data can actually determine the sub-resolution lateral variation of pore fluid properties within a reservoir. Generally, the relationship between the upscaled elastic constants and the effective fluid bulk modulus at the seismic scale is not the same as the relationship as that of the reservoir scale. However, the seismic-scale dependence of the undrained elastic constants on the pore-fluid properties of heterogeneous composites can be quantified using a Gassmann-type relation with the specific fluid bulk modulus that accounts for the pore fluid properties, the spatial arrangement of saturated patches, and the amount of upscaling.

The seismic-scale dependence of poroelastic constants upon the effective fluid bulk modulus is revealed by both physical modeling and numerical simulation experiments. A deeper understanding of this relationship will allow us to improve the estimates of petrophysical properties using the seismic-scale elastic constants.

## Methods

### Physical modeling

The physical modeling system used in this study mainly consists of the transducers (the transmitting and receiving instruments), the main control computer (signal generation and acquisition), the position controlling unit, and a large water tank (Supplementary Fig. S1). The piezoelectric transducers operating as the sources and receivers are homemade probes, with a length of 10 cm, a diameter of 2.5 mm, and a frequency range of 100−600 kHz. The gantry system can adjust the position of transmitting and receiver transducers in three dimensions, and has a positioning precision of 0.05 mm. Movement of transducers is controlled by high precision stepping motors. A data acquisition program written for signal control and acquisition runs on the desktop control computer and enables flexibility with respect to the source-receiver configuration. In the experiment, the positions of the centers of the source and receiver transducers are manually set to the origin of the physical model. These positions are stored in the acquisition program, which directs all subsequent positioning of the source and receiver. To conduct physical modeling experiments, a seismic wavelet is produced by a source transducer and the P-wave reflected signal is collected by receiving transducers and stored in computers, like in a conventional seismic recording. The physical modeling data can then be processed and inverted into the seismic-scale elastic variables.

## Supplementary information

Supplementary information
