# Supplementary material for: Influence of upscaling on identification of reservoir fluid properties using seismic-scale elastic constants
Source: Sci Rep. 2019 Sep 10;9:13056. doi: 10.1038/s41598-019-49559-2 (PMC6737066; doi:10.1038/s41598-019-49559-2)
Supplement: Supplementary file 1 — Supplementary information [file 41598_2019_49559_MOESM1_ESM.pdf]

Supplementary information for

Influence of upscaling on identification of fluid properties of reservoirs using seismic-scale elastic constants

Shengjie Li[1], Daxing Wang[2], and Mengbo Zhang[2]

[1]China University of Petroleum (Beijing), State Key Laboratory of Petroleum Resources and Prospecting, CNPC Key Laboratory of Geophysical Exploration, Beijing, China.

[2]Exploration and Development Research Institute of PetroChina Changqing Oil Field Company, Xi′an 710018, China; National Engineering Laboratory for Exploration and Development of Low-Permeability Oil & Gas Fileds, Xi′an 710018, CHina

Physical modeling experiment was carried out using a large physical modeling facilities at China University of Petroleum. Reflection experimental techniques was used to collect seismic data over a small-scaled physical model (As shown in Figure S1a). The physical model was constructed using 19 layers made by synthetic materials (as shown in Figure S1b). Before the construction of the physical model, a series of synthetic materials were manufactured using different stuff with different volume ratios, and the velocities of these synthetic aggregates were then measured using ultrasonic transmitting methods. The synthetic aggregate with specific components was identified for each layer based on the matching of the measured velocities and design parameters of the model. The details of the sample preparation, experimental steps, and uncertainty analysis of these synthetic materials are descripted in following section.

Sample Preparation and Description

There are two kinds of synthetic materials used for building the physical model, i.e., impermeable mixtures of epoxy resin, silicone rubber, and talcum powder with different proportions (R-S mixtures), and porous mixtures of quartz grains and resin with fixed volume ratios(S-R mixtures). Epoxy resin was prepared from industrial epoxy resin (E51), and quartz grains was prepared from industrial grade stone beads with average diameter range from 50 μm to 150 μm. These synthetic materials were manufactured in stainless steel mold box with a size of 70×70×70 mm, by stepwise filling and pressing of small portions at the same load to

achieve a homogeneous compaction. The composites were uniaxially pressed with pressures up to 4MPa at room-temperature. Cuboid samples of 70×70×50 mm were made through polishing the composites using a numerical control machine to ensure the smooth finish and parallelism of the sample surface.

Experimental Procedure and Results

Physical properties of the model were precisely measured. For all layers, these include the thickness of layers, *P*- and *S*-wave velocities and densities. Velocities of all layers were measured by a benchtop ultrasonic transmitting system. Additional parameters, such as porosity, were also tested on the synthetic porous materials.

The ultrasonic experimental system includes a TK-DPO3102 digital oscilloscope, CTS-8077 PR ultrasonic pulse emitter, Olympus V1012, V150 broadband ultrasonic transducers with dominant frequency of 0.25 MHz, as shown in Figure S2. The diameter of transducers was 38 mm. In measuring, the sample was placed between transmitting and receiving transducers, homemade honey was used as the coupling materials and transducers were pressed to improve the coupling between sample and transducers. We obtained the traveltime T_0 of the testing system by directly joining the transmitting and receiving transducers. Then, we subtracted T_0 from the total traveltime in the sample and divided the difference by the travel distance to get the *P*- and *S*- wave velocities of the samples.

The synthetic samples were measured in directions both parallel to and normal to the compression axis. *P*- and *S*-wave transmission signals were monitored and recorded using the digital oscilloscope at 9 test points in each direction for a sample, and the transmission signals were stacked 9 times to produce stable waveforms. The difference in measured *P*- and *S*-wave for R-S mixtures was less than 0.5% and 0.6% respectively, and there is no significant difference between the measured velocities in the direction parallel to and normal to compression axis. However, a relatively large variation in measured velocities for synthetic porous materials was observed. To reduce the uncertainty, four synthetic porous samples with similar proportions (as shown in Figure S3a) were used for determining the average *P*- and *S*-wave velocities of the synthetic porous layers. The microstructure of the synthetic porous sample was shown in Figure S3b. Specimens were dried in an oven for 48 h, at temperatures

of 50-80 ^{O}C, Prior to any experiments. *P*- and *S*-wave velocities of the dried specimens were firstly measured following the above mentioned testing procedure. The specimens then were saturated by evacuating them in a pressure chamber, water into the chamber and pressurizing the chamber. Velocities for these saturated specimens, evaluating by observing the weight of the specimens does not change, were measured again. Densities of all materials were determined by measuring the volume and mass of each individual sample. The total porosity was measured in an intelligent HCP-200 porosity apparatus with accuracy of 0.5 pu. The measured result for the synthetic porous materials were listed in Table S1.

The physical model was manufactured with a layer-by-layer casting method. The B10 layer was firstly made using the synthetic materials, five points, four at corners and one at central of the layer, were marked for references, and the shape of the layer was measured using a numerical controlled morphology measuring instrument with accuracy of 0.01 mm, the measurements at the five points were stored. The T9 layer then was made and these marked points were measured in the same way, until all layers were manufactured. The thickness of each layer were determined from the difference in the mean value of the five measured points between adjacent layers. The measured parameters for all layers were listed in Table S2.

Uncertainty Analysis

Errors and data inconsistencies are inevitable in measurements. Parameters of the measurement for synthetic samples consist of porosity, density, P- and S-wave velocities of the sample at dried and water-saturated condition. We assume that the variance of the porosity and density of samples are 0.6%, which is in the lower end of errors in experimental results. Factors associate with the measurement errors of velocities for synthetic samples include sample geometry accuracy (e.g., the length accuracy and the end flatness of samples), the size of transducers, traveltime picking accuracy, and the heterogeneity of samples. For 70 mm long synthetic samples, if the picking error is about 10 ms, the error for samples with *P*-wave velocity of 2500 m/s is approximately 16 m/s, accordingly, the measurement error is less than 1%, this error in measured S-wave velocity is slightly larger than that of the former. On average, the estimated mean error for P- and S-wave velocities are 16.5 m/s and 21.2 m/s, respectively. Another source of error in measurements is sample heterogeneity, which makes

the physical parameters of the synthetic porous layer within the physical model inconsistent with those of synthetic samples. It is reasonable to assume that the measurement errors of samples is random. Most of the errors attributable to the disturbance in measured porosity and velocities can be reduced, since upscaling essentially is an average of those variables of layers that composed of the package.

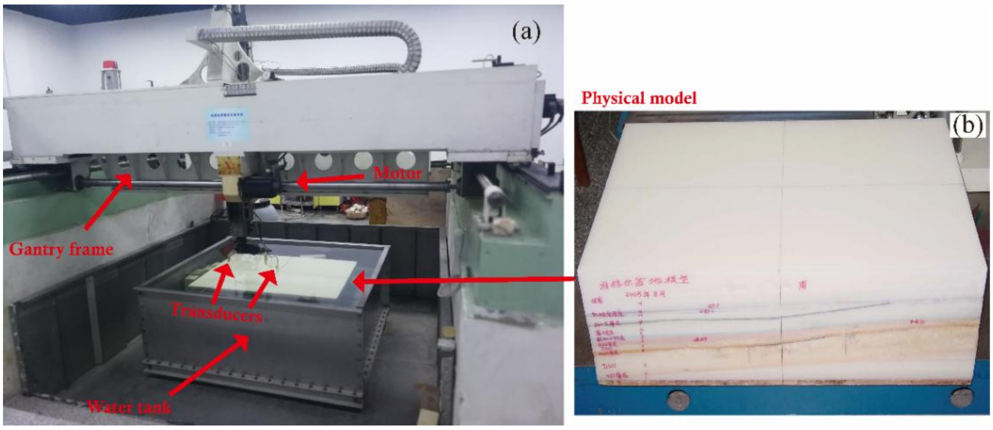

Figure S1. The physical modeling system (a) used in this study with the three-dimensional physical model (b).

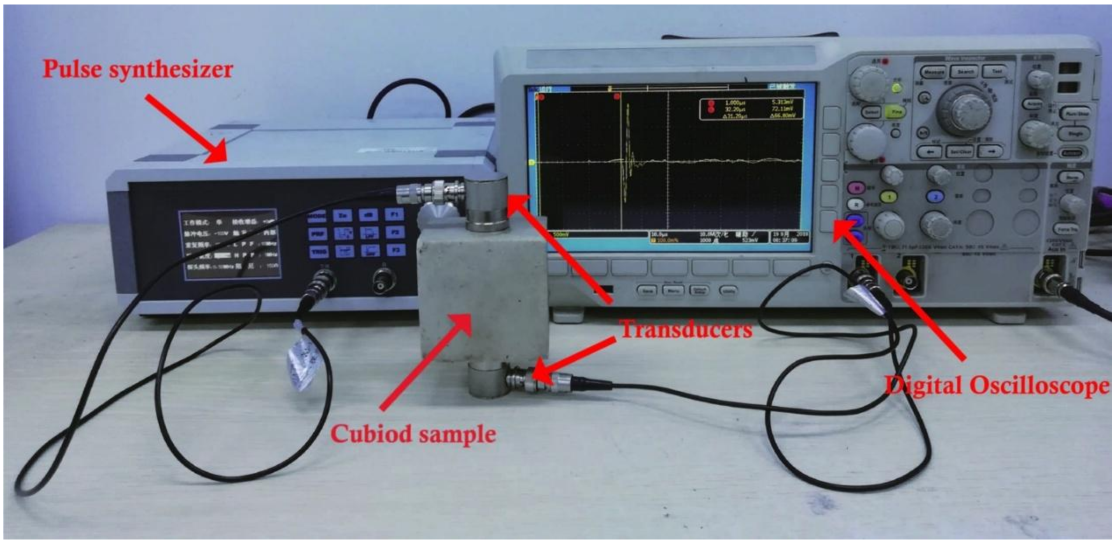

Figure S2. The bench-top ultrasonic measurement system used for *P*- and *S*-wave velocities measurements.

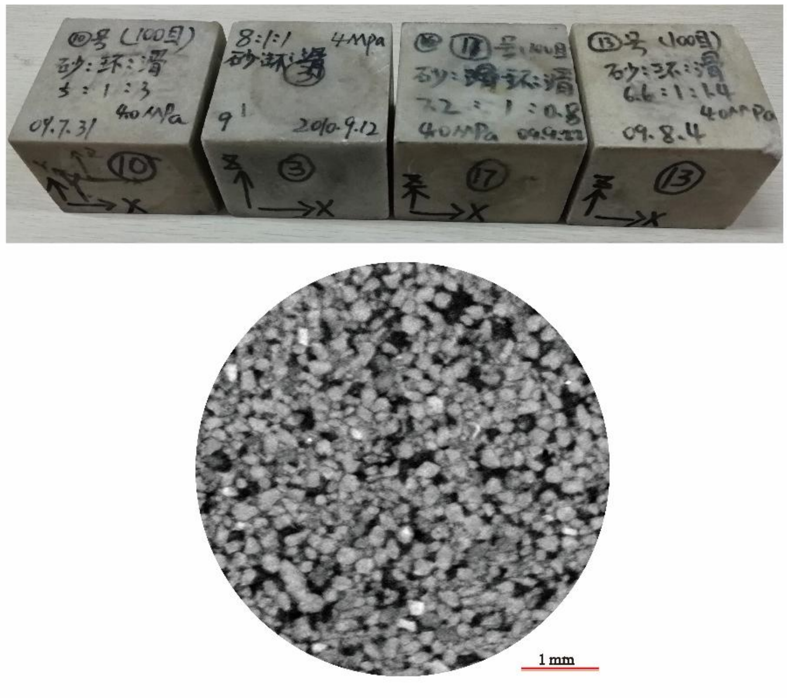

Figure S3. The synthetic cuboid samples (a) and their microstructures (b) produced by the micron CT imaging technique.

Table S1 Measured Velocities for synthetic sandstones With the Standard Deviations

		Dry Condition					Saturated Condition				
Sample	φ(%)	ρ(g/cm^3)	V_{ph}+σ	V_{pv}+σ	V_{sh}+σ	V_{sv}+s	ρ(g/cm^3)	V_{ph}+σ	V_{pv}+σ	V_{sh}+σ	V_{sv}+σ
S-3	23.8	1.13	2667.2+38	2572.8+31	1433.4+30	1410.6+34	1.37	2673.5+36	2594.5+33	1344.7+24	1331.3+31
S-7	23.4	1.13	2626.8+31	2549.2+40	1434.2+28	1405.8+29	1.37	2663.6+29	2600.4+28	1323.5+17	1302.5+24
S-8	24.1	1.12	2535.6+45	2490.4+38	1294.4+30	1281.6+19	1.37	2547.2+30	2506.8+35	1291.1+37	1280.9+26
S-10	24.7	1.11	2522.2+33	2437.8+36	1251.5+26	1236.5+27	1.36	2548.2+30	2487.8+36	1136.5+19	1127.5+35

Table S1. The physical properties of the synthetic sandstones.

Table S2 Measured Phyiscal Properties of Each Layer[a]

Layers	Thickness(mm)	Materials	Density(g/cm³)	Vp(m/s)	Vs(m/s)
B1	83.4	R-S Mixtures	1.15	2363	1227
T1	5.6	R-S Mixtures	1.19	2469	1247
B2	9	R-S Mixtures	1.15	2365	1230
T2	5.6	R-S Mixtures	1.19	2480	1250
B3	8.4	R-S Mixtures	1.15	2680	1322
T3	5	R-S Mixtures	1.16	2580	1291
B4	7.6	R-S Mixtures	1.19	2690	1326
T4	3.2	R-S Mixtures	1.19	2590	1295
B5	12.6	R-S Mixtures	1.21	2695	1325
T5	3	R-S Mixtures	1.03	2253	1106
B6	16.2	R-S Mixtures	1.21	2740	1338
T6	6	R-S Mixtures	1.11	2460	1247
B7	11.6	R-S Mixtures	1.20	2740	1342
T7	2.85	S-R Mixtures(dry)	1.12	2551	1342
		S-R Mixtures(Wet)	1.36	2572	1266
B8	16.4	R-S Mixtures	1.21	2742	1385
T8	3.35	S-R Mixtures(dry)	1.12	2551	1342
		S-R Mixtures(Wet)	1.36	2572	1266
B9	58.4	R-S Mixtures	1.22	2745	1345
T9	5.6	R-S Mixtures	1.14	2332	1203
B10	33.8	R-S Mixtures	1.19	2748	1348

[a]R-S mixtures stand for the resin-silicone rubber mixtures. S-R Mixtures stand for the Sand beads-Resin mixtures.

Table S2. The physical properties of each layer.
